# Anticancer Secondary Metabolites Produced by Fungi: Potential and Representative Compounds

**DOI:** 10.3390/ijms27010101

**Published:** 2025-12-22

**Authors:** Carlos García-Estrada, Carlos Barreiro, Juan F. Martín

**Affiliations:** 1 Departamento de Ciencias Biomédicas, Facultad de Veterinaria, Universidad de León, Campus de Vegazana s/n, 24007 León, Spain; 2 Instituto de Biomedicina (IBIOMED), Universidad de León, Campus de Vegazana s/n, 24007 León, Spain; 3 IBIOLEÓN (Instituto de Investigación Biosanitaria de León), Hospital de León, Altos de Nava s/n, 24071 León, Spain; c.barreiro@unileon.es; 4 Área de Bioquímica y Biología Molecular, Departamento de Biología Molecular, Facultad de Veterinaria, Universidad de León, Campus de Vegazana s/n, 24007 León, Spain; 5 Instituto de Biología Molecular, Genómica y Proteómica (INBIOMIC), Universidad de León, Campus de Vegazana s/n, 24007 León, Spain; 6 Área de Microbiología, Departamento de Biología Molecular, Facultad de Ciencias Biológicas y Ambientales, Universidad de León, Campus de Vegazana s/n, 24007 León, Spain

**Keywords:** fungi, secondary metabolites, antitumor, anticancer, clavaric acid, andrastins, fumitremorgin C, paclitaxel

## Abstract

Cancer remains one of the leading causes of death worldwide, and resistance to conventional therapies underscores the need for the discovery of novel antitumor agents. The ongoing search for novel natural sources offers promising avenues for discovering unique anticancer compounds with new mechanisms of action. One of these natural sources is represented by fungi, a prolific group of endophytic and non-endophytic eukaryotes able to produce bioactive secondary metabolites, many of which exhibit potent antitumor properties. These natural compounds display diverse chemical structures including polyketides, terpenoids, alkaloids, amino acid-derived compounds, phenols, etc. Their mechanisms of action are equally varied, ranging from induction of apoptosis and cell cycle arrest to inhibition of angiogenesis and metastasis. In this review we describe some potential antitumor compounds of fungal origin, together with the characteristics and biosynthesis of three representative types of antitumor compounds produced by filamentous fungi: squalene-derived sterol-type antitumor agents, prenylated diketopiperazine antitumor metabolites and meroterpenoid antitumor compounds. The ongoing scientific debate regarding the presence of paclitaxel biosynthetic genes in fungi is also discussed. As drug resistance remains a challenge in cancer therapy, fungal compounds offer a valuable reservoir for the development of new chemotherapeutic agents with novel modes of action.

## 1. Introduction

Canonical cell growth is the result of cell division, which is a highly regulated process. Abnormal or uncontrolled cell growth and division may lead to cancerous (malignant) tumors and neoplasms, or to non-cancerous (benign) tumors. Their spread into neighboring tissues, or their travel to distant sites in the body, result in new tumors (metastases), which are the primary cause of cancer-related mortality [1,2].

The National Cancer Institute (NCI), the United States federal government’s principal agency for cancer research and training, defines cancer as a “disease in which some of the body’s cells grow uncontrollably and spread to other parts of the body” (https://www.cancer.gov/about-cancer/understanding/what-is-cancer, accessed on 24 November 2025). This multifactorial disease results from genomic mutations in various genes regulating critical cellular functions, including transcription factors, tumor suppressor genes, cell surface receptors, oncogenes, kinases and phosphatases. These mutations can arise from diverse sources, such as: (i) inheritance; (ii) aging; (iii) environmental factors (e.g., ionizing radiations such as X-rays and ultraviolet light); (iv) lifestyle choices (e.g., smoking and alcohol) [3].

Nowadays, cancer is a leading cause of death worldwide [4]. According to data from the World Health Organization (WHO) via the International Agency for Research on Cancer (IARC), in 2022 there were 19.9 million new cases of cancer globally, with 9.7 million deaths. The highest incidence of cancer in 2022 was observed in: (i) lung (2.48 million); (ii) breast (2.29 million); (iii) colorectum (1.92 million); (iv) prostate (1.46 million); and (v) stomach (0.96 million). The most common causes of cancer death in 2022 were: (i) lung (1.82 million); (ii) colorectum (0.90 million); (iii) liver (0.76 million); (iv) breast (0.66 million); and (v) stomach (0.66 million) (https://gco.iarc.fr/today/en/fact-sheets-cancers, accessed on 24 November 2025).

Evidence of cancer dates back thousands of years (Smith and Ebers papyri) (https://training.seer.cancer.gov/disease/history/, accessed on 24 November 2025). Nowadays, the stage and type of cancer determine the prescribed treatment, which can range from traditional therapies (e.g., surgery, chemotherapy or radiotherapy) to newer approaches, including hormone-based therapies, gene-editing techniques, light-activated drugs (photodynamic therapy), molecularly targeted therapeutic agents, and immunotherapy [5]. Traditionally, most chemotherapeutic anticancer drugs are natural products or synthetic derivates targeting specific cellular functions. Medicinal plants used in ancient Asian cultures as remedies for various disorders, including cancer, lie at the heart of the drug discovery process [5,6,7,8]. In addition, microorganisms such as bacteria and fungi have also served as a source of pharmacological agents likely due to their lifestyle in multispecies, dense communities, where fluctuations in nutrient availability drive the production of a variety of survival tools (toxins, siderophores, antibiotics, etc.) for their ecological “warfare” [9,10]. Compounds such as alkaloids, flavonoids, terpenoids, polyphenols, and saponins are present in plants and different microorganisms, and it has been reported that more than 60% of current cancer drugs, such as vinca alkaloids, camptothecin and paclitaxel, are derived from natural sources [7,11,12,13].

Actinobacteria and filamentous fungi are the most important producers of secondary metabolites, which exhibit potent biological activities relevant to human, animal and plant health [14,15]. Thus, a single fungal species can produce more than one hundred secondary metabolites, resulting from the activation of several dozen biosynthetic gene clusters [14]. Fungi from diverse origins (terrestrial, marine, endophytes and non-endophytes) are producers of several clinical drugs (antibacterials, antifungals, cholesterol-lowering compounds, or immunosuppressants [16,17,18,19]), and have proven to be a crucial source of bioactive compounds with anticancer properties [11]. They thus serve as a rich reservoir for discovering novel anticancer drugs, highlighting the importance of fungi in cancer therapy research. In this review, we focus on fungal-derived anticancer compounds and describe some of the key biosynthetic pathways involved in the production of the most representative compounds. We also provide information on the preclinical and clinical status, pharmacokinetic properties, toxicity and production constraints.

## 2. Fungal Secondary Metabolism as a Source of Bioactive Compounds

Fungi represent one exceptional source of bioactive compounds. These eukaryotic heterotroph microorganisms with sexual and asexual reproduction can occur as free-living or symbiotic yeasts, molds, mushrooms, lichens or zoosporic forms in every ecosystem of the world [20] and have been classified into nine recognized phyla [21].

Fungi stand out due to their extraordinary chemical diversity and the variety of their secondary metabolites, many of which have given rise to crucial medicines [22,23,24]. Fungal secondary metabolites derive from specialized pathways catalyzed by enzymes whose coding genes are arranged in gene clusters, thereby facilitating their joint regulation. These genomic regions encode key biosynthetic enzymes, oxidoreductase enzymes, transporters, and specific regulators [25,26]. Many fungal genomes contain abundant latent biosynthetic gene clusters that can be activated by epigenetic regulation, revealing new compounds with anti-inflammatory, anticancer and antimicrobial potential [26,27]. A survey across nearly 11,598 fungal genomes identified approximately 293,926 biosynthetic gene clusters, grouped into 26,825 gene cluster families, with less than 1% associated with characterized compounds, thus highlighting a vast, untapped biosynthetic potential [28].

The immense chemical diversity of fungal secondary metabolites encompasses multiple structural classes such as polyketides (PKs), non-ribosomal peptides (NRP), ribosomally modified peptides, terpenoids, alkaloids, and hybrid metabolites [29]. In fungi, the shikimate pathway—an essential route for synthesizing aromatic compounds—is conserved and operates as a multifunctional enzymatic system known as the AROM complex, catalyzing multiple steps from 3-deoxy-D-arabino-heptulosonate-7-phosphate to shikimate 3-phosphate and onward to chorismate [30]. From chorismate, fungi branch into producing aromatic amino acids (phenylalanine, tyrosine, tryptophan), which serve as precursors for diverse secondary metabolites including pigments, alkaloids, and polyphenols [31].

PKs (e.g., griseofulvin, fumonisin) are the most numerous and diverse class of secondary metabolites in fungi and are constructed from acetyl-CoA and malonyl-CoA units by PK synthase (PKS) enzymes, mainly large multidomain iterative type I PKS [32,33,34,35]. NRP (e.g., β-lactam antibiotics, cyclosporin A) are produced by large modular and multidomain enzymes called NRP synthetases (NRPS), independently of the ribosomal machinery [36,37,38]. Recent studies have revealed that fungi harbor a fascinating repertoire of ribosomally synthesized and post-translationally modified peptides (RiPPs) (e.g., α-amanitin, phalloidin) with unique structures and potent biological functions [39,40]. Terpenes (e.g., carotenoids, gibberelllins) are produced by terpene synthases from isoprenoid precursors (isopentenyl pyrophosphate and its isomer dimethylallyl pyrophosphate) formed primarily via the mevalonate pathway, thus giving rise to monoterpenes (C10), sesquiterpenes (C15), diterpenes (C20), and bigger structures that are further modified by other enzymes, such as prenyltransferases or cyclases [41,42,43]. Alkaloids (e.g., ergotamine, roquefortine C) are nitrogen-rich secondary metabolites that exhibit multiple biological activities, such as cytotoxic, antibacterial, antifungal, antioxidant, antiviral, anti-inflammatory, and antifouling [44,45,46,47]. Fungal siderophores (e.g., fusarinine C, rhizoferrin) are low-molecular-weight secondary compounds with high affinity for ferric iron (Fe^3+^) that are biosynthesized by NRPS or NRPS-independent pathways and then secreted to capture iron and transport it back to the cell by specialized transporters [48,49,50,51,52]. Finally, some fungal hybrid metabolites contain domains synthesized by different routes, such as PK-amino acid metabolites formed by iterative PKS-NRPS (e.g., cyclopiazonic acid, fusarin C [53,54,55]) and meroterpenoids formed after the transfer of farnesyl pyrophosphate (C15) to PK derivatives (e.g., mycophenolic acid, fumagillin [56]).

## 3. Potential and Representative Anticancer Compounds Derived from Fungi

The structural and functional richness of fungal secondary metabolites reflects the complexity of the fungal biosynthetic pathways. As research advances through the study of biosynthetic gene clusters and the use of genomic and biotechnological tools, endophytic and non-endophytic fungi are increasingly recognized as a largely untapped natural resource with immense potential in cancer therapy, making them a valuable source for the discovery of new anticancer drugs.

Endophytic fungi [57] are a rich and promising source of metabolites with diverse bioactivities, including antitumor activity [58,59,60]. From the discovery of paclitaxel (Taxol^®^) in *Taxomyces andreanae* [61], although still controversial [62], to the identification of new compounds such as camptothecin, complex epoxides, and difunctional quinones, research has revealed multiple avenues for the development of anticancer drugs from these microorganisms. On the other hand, non-endophytic fungi represent a broad group of either pathogenic organisms that cause adverse effects in plants, animals, and humans [63], or saprophytic fungi that play a key role in terrestrial ecosystems due to their ability to decompose dead organic matter and recycle nutrients facilitating their efficient distribution throughout the soil system. These fungi, apart from performing essential ecological functions [64], are also a significant source of antitumor compounds [11,65,66,67].

**Figure 1:**
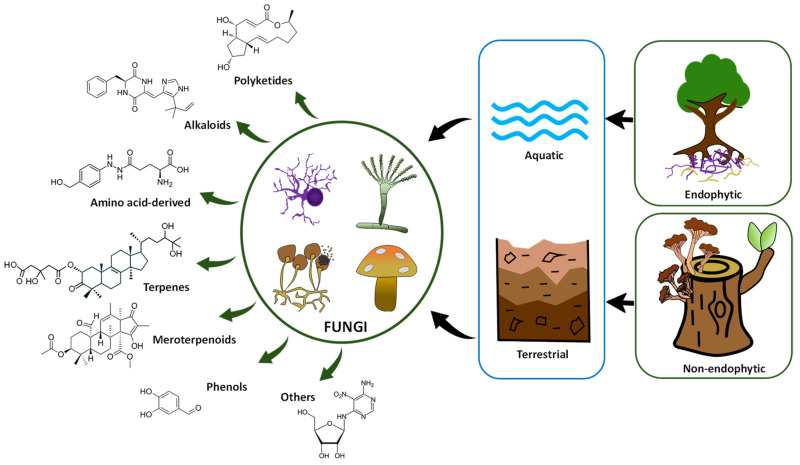
Schematic representation of the major antitumor chemical classes produced by fungi.

Some recent articles have deeply reviewed the terrestrial and marine endophytic and non-endophytic fungal compounds with potential anticancer activity (see, for example, [11,58,59,60,68]). This section provides examples of potential and representative anticancer compounds produced by fungi (Figure 1).

### 3.1. Polyketides (PKs)

Fungal PKs represent a rich source of antitumor agents with diverse scaffolds and mechanisms. Some PKs from fungi with documented anticancer properties are indicated below.

**Figure 2:**
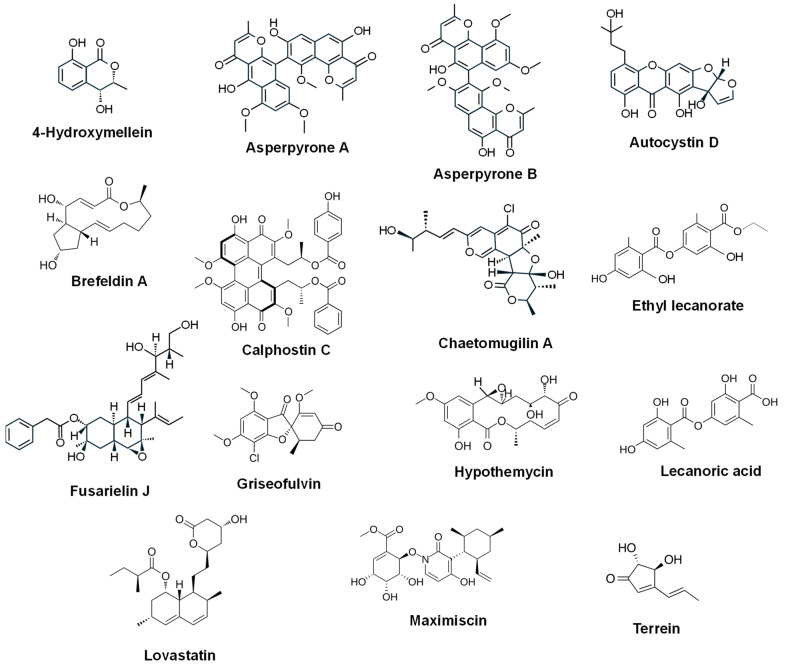
Chemical structure of fungal PKs with antitumor activity.

Statins (lovastatin, compactin/mevastatin and simvastatin), cholesterol-lowering PK-derived compounds, are produced by different fungi, such as *Aspergillus terreus*, *Penicillium citrinum* or *Monascus ruber* [69]. Beyond their lipid-lowering effects, statins exhibit anticancer properties by inducing apoptosis, inhibiting tumor cell proliferation, and impairing angiogenesis. For example, **lovastatin** (Figure 2), in addition to inhibit hydroxy-3-methylglutaryl (HMG)-CoA reductase, crucial for cholesterol synthesis, has been shown to induce apoptosis, cause cell cycle arrest across diverse tumor types (including breast, liver, cervical, lung, and colon cancers), enhance chemosensitivity to chemotherapeutic drugs and strengthen their therapeutic effect [70]. Lovastatin, mevastatin and simvastatin were able to inhibit the growth, cell migration and invasion of different melanoma cells [71]. In recent decades, statins have garnered significant attention for their potential anticancer effects, and several preclinical and clinical studies have confirmed the beneficial antitumor activities of this type of PKs [72].

The aromatic PKs 4,5-dihydroxy-6-(6′-methylsalicyloxy)-2-hydroxymethyl-2-cyclohexen-l-one, gentisyl alcohol and 6-(hydroxymethyl)benzene-1,2,4-triol were produced by the pathogenic fungus *Epicoccum sorghinum* and showed cytotoxicity against A549, HepG2, and MDA-MB-231 tumoral cell lines [73]. Other aromatic PKs include the dimeric naphthopyrones **asperpyrone A** (active against SK-OV-3 human ovarian cancer cells) and **asperpyrone B** (active against PANC-1 human pancreatic cancer cells) (Figure 2), which are produced by *Aspergillus* sp. XNM-4, derived from marine algae *Leathesia nana* (Chordariaceae) [74].

The 16-membered macrolide **brefeldin A** (Figure 2) is produced by various fungi (*Paecilomyces*, *Alternaria*, *Curvularia*, *Penicillium*, *Phyllosticta*) and has been described as cytotoxic for different tumoral cell lines, such as HL-60, KB, HeLa, MCF-7 and Spc-A-1 [75,76,77]. This compound interferes with the normal maintenance of the Golgi membrane [78]. **Terrein** (4,5-dihydroxy-3-[(E)-1′-propenyl]-2-cyclopenten-l-one, C_8_H_10_O_3_) (Figure 2), which was isolated from *A. terreus* in 1935 [79], displays antitumor functions on different cancer types, such as ovarian [80], esophageal [81] and colorectal [82]. The chlorinated antifungal metabolite **griseofulvin** (Figure 2), was first isolated by the soil fungus *Penicillium griseofulvum*, although it is also synthesized by other ascomycete fungi [83]. This compound inhibits tumor growth and several forms of cancer cell proliferation by microtubule dynamics suppression and mitotic arrest [84,85], providing significant antitumor activity against cervical cancer, breast cancer, non-small-cell lung cancer, colorectal cancer, and adrenocortical cancer [86]. **Calphostin C** (Figure 2) is one of the calphostins, a group of perylenequinone compounds isolated from the fungus *Cladosporium cladosporioides* [87]. Under light conditions, it strongly inhibits proliferation of both malignant and low-grade glioma cell lines, as well as neonatal rat astrocytes [88]. Calphostin C also induces cytotoxicity in breast cancer cells through induction of endoplasmic reticulum stress [89], triggers calcium-dependent apoptosis in various acute lymphoblastic leukemia cells [90] and shows growth inhibition cervical cancer HeLa-S3 and breast cancer MCF-7 cell lines [91]. **Chaetomugilins** (Figure 2) are a family of azaphilone-type secondary metabolites with a characteristic oxygenated bicyclic pyranoquinone core produced by the fungus *Chaetomium globosum*, often isolated from marine fish (*Mugil cephalus*)-associated strains [92]. These compounds showed cytotoxicity against different tumor cell lines [92,93]. **Austocystin D** (Figure 2) is a secondary metabolite originally isolated over three decades ago from maize meal cultures of *Aspergillus ustus* (now often reclassified within the *Aspergillus* section Usti) [94] that is cytotoxic against a subset of cancer cells [95,96]. **Hypothemycin** (Figure 2) is a macrocyclic PK that belongs to the β-resorcylic acid lactone family that was primarily isolated from *Hypomyces subiculosus* (also referenced historically as *Hypomyces trichothecoides*) [97]. It exhibits promising anticancer activities, primarily through its role as a covalent inhibitor of protein kinases [98] and demonstrated antitumor activity in mouse tumor xenograft models [99]. **Fusarielin J** (Figure 2) is produced by the endophyte *Fusarium tricinctum*, isolated from rhizomes of *Aristolochia paucinervis*, and showed cytotoxicity against the human ovarian cancer cell line A2780 [100]. *Phoma* sp., an endophytic fungus from the medicinal plant *Cinnamomum mollissimum* produces **4-Hydroxymellein** (Figure 2), which showed strong inhibitory activity against P388 murine leukemic cells [101]. Through a crowdsourcing program, the unique PK–shikimate–NRPS hybrid compound, **maximiscin** (Figure 2), was obtained from *Tolypocladium* sp. via chemical epigenetic induction, culture medium variation, and bacterial co-culture strategies. This compound demonstrated cytotoxic effects in vitro across various cell types and exhibited antitumor activity in vivo using a UACC-62 xenograft cancer model [102]. Maximiscin also showed selective cytotoxic activity against MDA-MB-468 cells modeling the BL1 subtype of triple-negative breast cancer [103].

Several fungal depsides (acetyl-poly-malonyl-derived PKs) have been reported to have antitumor activity [104]. For example, **lecanoric acid** and **ethyl lecanorate** (Figure 2), which were purified from *Claviceps purpurea*, exerted antitumor effects on the human cancer cell lines HepG2 (liver) and CCF-STTG1 (astrocytic-like) [105]. Other examples are represented by CRM646-A and CRM646-B, both discovered from *Acremonium* sp. and exhibiting potent anti-metastatic capacity against B16-F10 melanoma cells [106].

### 3.2. Amino Acid-Derived Molecules

#### 3.2.1. Diketopiperazine (DKP) Alkaloids: Representative Molecules and Biosynthesis

DKPs are modified cyclic dipeptides, and many natural DKPs have been characterized over the last decades. They share the common feature of a six-membered DKP ring formed by a double condensation between the carboxyl group of amino acid 1 and the amino group of amino acid 2, and vice versa. The most frequent amino acids involved in DKP formation are tryptophan, proline, histidine, phenylalanine, and alanine. The resulting DKP ring is a very stable, planar structure that is resistant to protease degradation [107].

**Figure 3:**
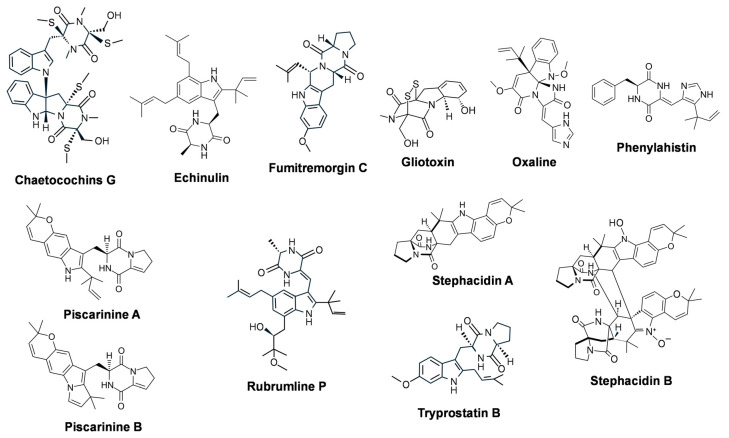
Chemical structure of fungal DKP alkaloids with antitumor activity.

In recent years, these compounds have attracted great interest due to their biological activities, particularly their ability to inhibit tumor cell growth [108]. The first antitumor DKP described was **phenylahistin** (Figure 3), produced by *A. ustus*, which is formed by the condensation of phenylalanine and dehydrohistidine [109]. In phenylahistin, the DKP ring is prenylated by a dimethylallyl diphosphate reverse prenyltransferase. Phenylahistin disrupts microtubule formation, thereby inhibiting tumor cell growth by interfering with tubulin polymerization [110], in a mode of action that is similar to that of the plant-derived compound paclitaxel (see below). Cytotoxic activity of phenylahistin was first observed in a screening program conducted by the Japanese Foundation for Cancer Research [109,110]. The antitumor activity of phenylahistin was later confirmed through studies on lung carcinoma cells and other tumor cell lines [111,112]. In addition, its synthetic derivative plinabulin has shown potent antitumor effects and has entered clinical trials [113,114,115]. Unfortunately, detailed studies on the biosynthesis of phenylahistin have not yet been reported.

DKPs exhibit a broad range of cytotoxic activities, but only a few have demonstrated selectivity against tumor cell lines. The most active compounds are typically those containing tryptophan combined with another aromatic or aliphatic amino acid within the DKP ring. A well-studied example is **tryprostatin B** (Figure 3), produced by *Aspergillus fumigatus* and other *Aspergillus* species. Tryprostatin B is formed through condensation of tryptophan with proline. In the initial biosynthetic step, these amino acids are condensed by a DKP synthetase to form brevianamide F. In a subsequent step, brevianamide F is prenylated by a reverse prenyltransferase, which introduces a dimethylallyl group at the C2 position of the tryptophan unit. Tryprostatin B, along with its methoxylated derivative tryprostatin E and the related compound fumitremorgin, displays potent activity against human tumor cell lines [116,117,118]. The fumitremorgins and verruculogen (see below) are biochemically related to tryprostatins. **Fumitremorgin C** (Figure 3) is biosynthesized by *A. fumigatus* and exerts a potent and selective inhibition of the breast cancer resistance protein (BCRP/ABCG2), an ABC transporter that contributes to multidrug resistance in several types of cancer cells [119,120]. Inhibitors of this protein were used in the screening of potential antitumor agents [121,122,123].

Comparative studies of prenylated versus non-prenylated derivatives of cyclo-(tryptophan-proline) or cyclo-(tryptophan-alanine) clearly demonstrated that antitumor activity is associated with the prenylated forms, while the non-prenylated DKPs show little or no activity [124]. Interestingly, changing the second amino acid (from proline to alanine) has little effect on activity, and different stereoisomers of tryptophan also show minimal impact. Since prenylation or palmitoylation often targets these molecules to vesicular or plasma membranes [125], it is plausible that their antitumor activity is related to their localization on animal cell membranes.

Other DKP alkaloids with antitumor activities produced by fungi are **Stephacidin A** and **stephacidin B** (dimeric form and more potent than stephacidin A) (Figure 3). They are produced in solid fermentation by *Aspergillus ochraceus* and show antitumor activity in cell lines such as the prostate testosterone-dependent LNCaP cells [126]. These compounds are also produced by the indoor mold *Aspergillus westerdijkiae* [127]. **Piscarinins A** and **B** (Figure 3) are synthesized most actively during the surface cultivation of the fungus *Penicillium piscarium* and exhibited cytotoxicity against the prostate cancer cell line LNCaP [128]. **Oxaline** (Figure 3), an O-methylated derivative of meleagrin isolated from *Penicillium oxalicum*, has been reported to inhibit tubulin polymerization, leading to cell cycle arrest at the M phase in Jurkat cells [129]. **Gliotoxin** (Figure 3), a biologically potent mycotoxin produced by *A. fumigatus* [130], *Neosartorya pseudofischeri* [131], *Trichoderma virens* [132] and *Dichotomyces cejpiii* [133] shows a characteristic redox-active disulfide bridge in the DKP core. In recent years, gliotoxin has garnered attention for its anticancer potential in breast cancer, with striking cytotoxic potency in tumoral cell lines such as MDA-MB-231, MDA-MB-468, and MCF-7 [134], in colorectal cancer, inducing apoptosis in several colorectal cancer lines [135], and in other cancer-derived cell types, including lung epithelial cells and chronic lymphocytic leukemia lines, via mitochondrial disruption, activation of Bax, caspases, and cytochrome c release [136]. **Rubrumline P** (Figure 3), was isolated from the marine sediment-derived fungus, *Aspergillus chevalieri*, and has cytotoxic activity against PANC-1 cancer cells [137]. **Chaetocochins G** (Figure 3), which was produced by the endophytic fungus *Chaetomium cochliodes* 88194, induces apoptotic cell death in MCF-7 breast cancer cells [138]. The endolichenic fungus *Aspergillus* sp., which was isolated from the lichen *Xanthoparmelia conspersa*, produces **echinulin** (Figure 3) and 8-hydroxyechinulin, both compounds showing antiproliferative activity against HT-29 cells [139].

**Figure 4:**
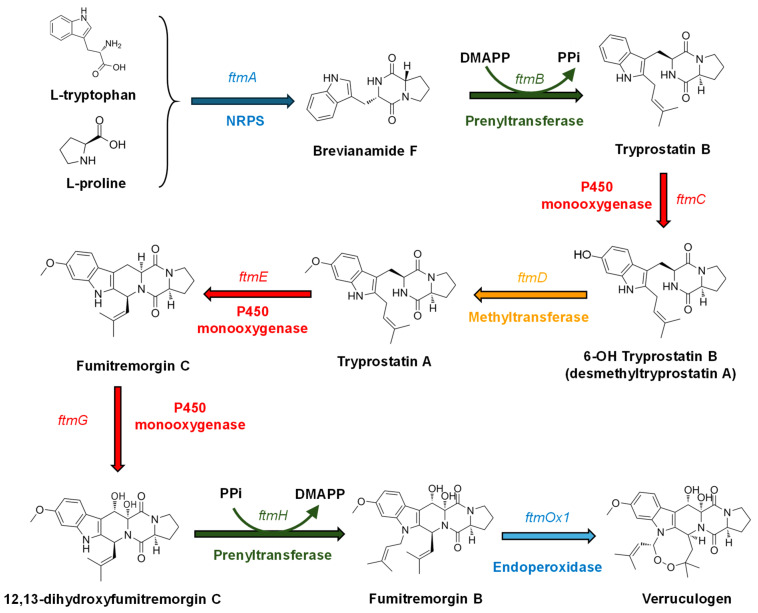
Representative DKP alkaloids biosynthetic pathway exemplified by fumitremorgin/verruculogen biosynthesis.

Fungal antitumor DKPs are synthesized by NRPS enzymes [140] that use ATP to activate substrate amino acids as aminoacyl-AMPs. They differ from bacterial DKPs cyclodipeptide synthases, which use aminoacyl-tRNAs rather than free amino acids [141]. The tryprostatin/fumitremorgin biosynthetic pathway is shown as an example (Figure 4). Early molecular genetic studies [142] identified the gene encoding the biosynthesis of brevianamide F, a precursor of tryprostatin B and fumitremorgins A, B, and C. The *ftmA* gene encodes the NRPS brevianamide F synthetase (dipeptide synthetase) and is part of the fumitremorgin gene cluster in *A. fumigatus* [143]. Sequence analysis revealed that FtmA has a dimodular ATC-ATC structure (A = adenylation domain, T = thiolation (peptidyl carrier protein) domain, and C = condensation domain). A similar dimodular ATC-ATC architecture is found in the roquefortine C dipeptide synthetase [47,144], suggesting conservation of this structure across fungal DKP synthetases. In the second step of the tryprostatin/fumitremorgin pathway, brevianamide F is prenylated by the prenyltransferase encoded by *ftmB*, which introduces a dimethylallyl group at the C2 of the tryptophan indole ring. Tryprostatin B is then converted into tryprostatin A in two steps: hydroxylation at C6 by the P450 monooxygenase encoded by *ftmC*, followed by methylation of the hydroxyl group by the methyltransferase encoded by *ftmD*, forming a methoxyl group [120]. Tryprostatins A and B are intermediates in a larger pathway that also yields fumitremorgins and verruculogen. Indeed, genome studies indicated that a large gene cluster containing genes for the biosynthesis of these different compounds is located on chromosome VIII of *A. fumigatus* [145].

Three oxygenases, one methyltransferase and two prenyltransferases encoded in genes of the fumitremorgin cluster have been characterized. The genes encoding P450 oxygenases—*ftmC*, *ftmE*, and *ftmG*—are part of the fumitremorgin–tryprostatin pathway: the protein encoded by *ftmC* is involved in the conversion of tryprostatin B to 6-hydroxy-tryprostatin B; the protein encoded by *ftmE* is involved in the key conversion of tryprostatin A to fumitremorgin C by catalyzing an oxidative ring closure between carbon C3 and N4 of the tryprostatin structure. This C3–N4 condensation is very unusual for a P450 oxygenase and is rarely observed in nature [120]. Finally, the protein encoded by *ftmG* is involved in the conversion of fumitremorgin C to 12,13-dihydroxyfumitremorgin C. The O-methyltransferase involved in the conversion of 6-hydroxy-tryprostatin B to tryprostatin A was characterized [146] by comparing this protein in *A. fumigatus* BM939 (a fumitremorgin-producing strain), with strain Af293, which is unable to produce the compound. Comparison of the methyltransferase amino acid sequences in both strains revealed a mutation in amino acid R202L, adjacent to conserved acidic residues and close to the enzyme’s active site. The arginine-to-leucine mutation renders the O-methyltransferase from *A. fumigatus* Af293 functional in vitro, but not in vivo.

In addition, the cluster contains two prenyltransferases encoded by the *ftmB* and *ftmH* genes (also named ftmPT1 and ftmPT2, respectively). The *ftmB* gene encodes the prenyltransferase involved in the transfer of dimethylallyl-PP to brevianamide F (described above) [147], whereas the second prenyltransferase is responsible for the final step in the formation of fumitremorgin B, a diprenylated compound. Notably, the prenyltransferase encoded by *ftmH* differs significantly from the brevianamide prenyltransferase encoded by *ftmB*, sharing only 30% amino acid identity. Biochemical studies using purified protein, obtained after cloning the *ftmH* gene and expressing it in *Saccharomyces cerevisiae*, demonstrated that the protein is an N-prenyltransferase that introduces the dimethylallyl group at the N1 position of the indole ring of 12,13-dihydroxyfumitremorgin C, forming fumitremorgin B [143]. It is known that distinct prenyltransferases introduce the dimethylallyl group at different positions of the indole ring during the biosynthesis of secondary metabolites, including roquefortine [25,47] and fumigaclavine, either in *A. fumigatus* [145,148] or in *Penicillium roqueforti* [149]. The diversity of prenyltransferases in the fumitremorgin gene cluster, as well as in other fungal clusters, provides tools for chemosynthetic production of novel DKP derivatives with antitumor activity [150]. For example, this can be achieved by combining various DKPs with well-characterized prenyltransferases.

The role of additional genes in the cluster, namely *ftmOx1*, remained obscure for many years. It was observed that this gene encodes an endoperoxidase that introduces two oxygen atoms into fumitremorgin B, converting this substrate into verruculogen by inserting an endoperoxide bond between two prenyl groups [151]. Ascorbic acid, Fe^2+^ and α-oxoglutarate are required cofactors for the reaction, as in other non-heme oxygenases. Therefore, the fumitremorgin pathway should be referred to as the fumitremorgin/verruculogen pathway. The molecular mechanism of the conversion of fumitremorgin B to verruculogen by the endoperoxidase FtmOx1 has been studied recently. It was observed that a mutation in residue 68 (Y68) of the endoperoxidase renders it unable to convert fumitremorgin B to verruculogen [152]. Recently, the X-ray crystal structure of the ternary complex FtmOx1–α-ketoglutarate–fumitremorgin B was obtained [153]. These authors found that binding of fumitremorgin compresses the active pocket, favoring the proximity of Y68 to the substrate and the active center, thus enabling endoperoxidase activity. This agrees with previous observations on enzyme inactivation by a Y68P mutation [152,154].

In recent years, important advances have been made in the study of fungal DKPs, for example, elucidation of the biosynthetic pathway of prenylated equinulin and isoequinulin in *Aspergillus ruber* [155], and the production of a cyclic Proline–Valine DKP by the marine yeast *Meyerozyma guilliermondii* [156]. However, the medical applications of these novel DKPs have not yet been studied in detail.

#### 3.2.2. Other Amino-Acid Derived Compounds

**Figure 5:**
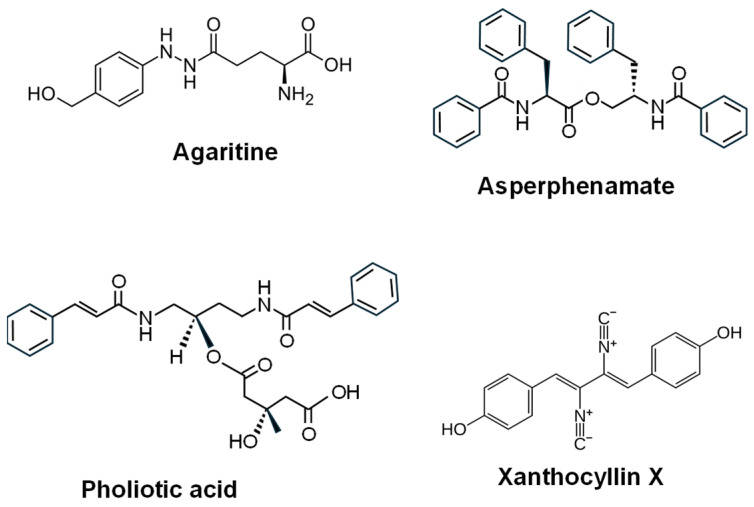
Chemical structure of some fungal amino acid-derived compounds with antitumor activity.

The L-tyrosine-derived isocyanide-bearing aromatic **xanthocillin X** (Figure 5) was first isolated from *Penicillium notatum* [157], but it can be biosynthesized by other filamentous fungi, such as *Penicillium commune* [158] or *A. fumigatus* [159]. It showed antiproliferative activity in glioma cell lines U251 and U87MG [160]. Some structural analogs have also provided promising anticancer activities [65,161].

The linear amino acid ester (NRPS-derived dipeptide ester) **asperphenamate** (Figure 5) is produced by different *Aspergillus* and *Penicillium* species [162,163,164]. Asperphenamate exhibited moderate inhibitory effects against several cancer cell lines, and BBP (N-benzoyl-O-(N′-(1-benzyloxycarbonyl-4-piperidiylcarbonyl)-D-phenylalanyl)-D-phenylalaninol), a solubility-enhanced asperphenamate analog, demonstrated antitumor effect on different cell lines and growth inhibition in MCF-7 breast cancer cells via JNK-dependent autophagy [65,165,166].

The amino-acid derivative **pholiotic acid** (Figure 5), isolated from the basidiomycete *Pholiota spumosa*, is a polyamine analogue of putrescin that significantly reduced the viability of human metastatic melanoma cell lines (M14 and A2058) by inducing intrinsic apoptosis [167]. **Agaritine** (Figure 5), a non-proteinogenic L-α-amino acid (a glutamic acid conjugated with phenylhydrazine), was isolated from *Agaricus blazei* mushroom extracts and exhibited potent antiproliferative activity against leukemic cells [168]. Certain mushrooms produce unique amino acid-modified alkynyl compounds. Examples include derivatives of aminohexynoic acid from *Tricholomopsis rutilans*, which exhibit anticancer, antiviral, and cholesterol-lowering activities [169].

Several macromolecules, like proteins and peptides obtained from different mushroom species, have shown promising anticancer effects. Some examples are mushroom lectins [170], protein-bound polysaccharides (polysaccharide-K (PSK) or krestin and polysaccharopeptide (PSP) isolated from *Coriolus versicolor* or *Lentinula edodes*) [171], low-carbohydrate proteins, including enzymes (marmorin, velin, pleureryn, pleuturegin, etc.), ubiquitin-like peptides (CULP, RBUP, PSULP, UbcA1, etc.), and fungal immunomodulatory proteins (Lingzhi-8, PCiP, APP, TVC, etc.), among others [172]. Specific bioactive peptides isolated from mushroom mycelia or fruiting bodies have displayed cytotoxic and pro-apoptotic effects. For example, a peptide with sequence S-L-S-L-S-V-A-R derived from *Morchella* species was reported to reduce tumor cell proliferation via mitochondrial-mediated apoptosis [173].

### 3.3. Terpenes

Terpenes are hydrocarbons built from repeating isoprene units (C_5_H_8_) whose structures range from simple linear chains to complex cyclic arrangements. According to the number of isoprene units, they are classified as monoterpenes (two units), sesquiterpenes (three units), diterpenes (four units), sesterterpenes (five units), triterpenes (six units), etc. The diversity in their carbon skeletons leads to a wide variety of chemical and biological properties, including antitumor activity.

#### 3.3.1. Sesquiterpenes

**Figure 6:**
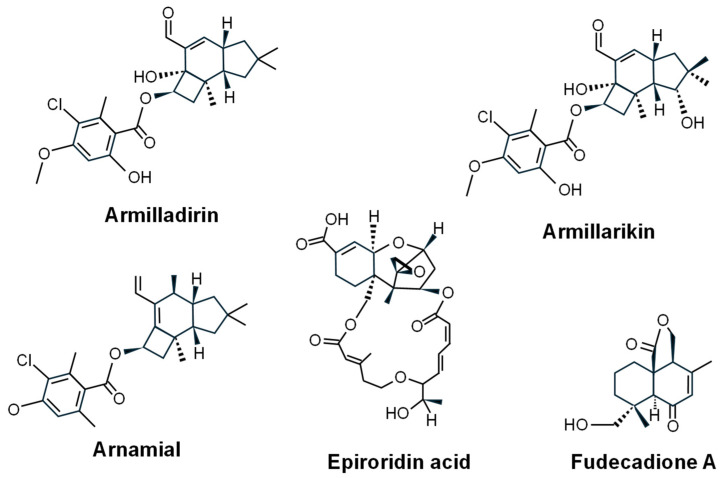
Chemical structure of fungal sesquiterpene compounds with antitumor activity.

**Fudecadione A** (Figure 6) is a drimane-type sesquiterpene isolated from the soil fungus *Penicillium* sp. BCC 17468 [174] and the marine-derived fungal strain *Penicillium* sp. TW58-16 [175]. It exhibited anticancer activity against MCF-7, KB, and NCI-H187 tumoral cells [174].

Several sesquiterpene aryl esters (e.g., arnamial, armillaridin, armillarikin) are produced by the saprophytic mushroom species *Armillaria mellea* [66]. **Arnamial** (Figure 6) exhibits cytotoxic against human colon, breast, leukemia cell lines [176]. **Armillaridin** (Figure 6) was active in leukemia cell lines [177] and also demonstrated radiosensitizing effects in esophageal carcinoma cells [178]. **Armillarikin** (Figure 6) induced apoptosis in leukemia and hepatocellular carcinoma lines [179,180].

**Epiroridin acid** (a sesquiterpenoid macrolide) (Figure 6) isolated from the endophytic *Myrothecium roridum* A553 exhibited strong cytotoxicity against different human cancer cell lines (MCF-7, SF-268, NCI-H460, HepG2) [181].

#### 3.3.2. Paclitaxel and Other Diterpenes

**Figure 7:**
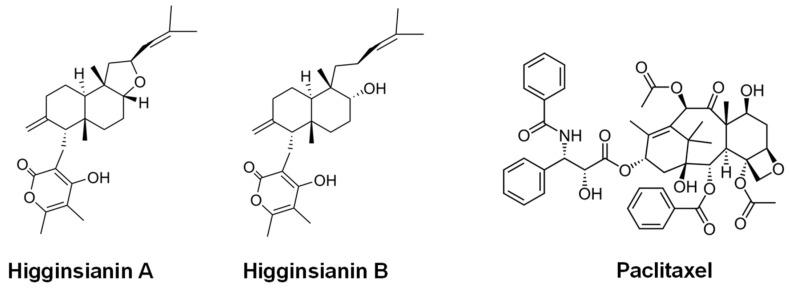
Chemical structure of paclitaxel and other fungal diterpenes with antitumor activity.

Paclitaxel (Taxol^®^) (Figure 7) was discovered in 1971 in the bark of the Pacific yew *Taxus brevifolia* [182]. This compound has a very complex chemical structure with eleven chiral centers. Taxol^®^ is one of the most potent natural products for the treatment of solid human tumors, particularly breast and ovarian cancer [182,183]. It acts by binding to tubulin and inhibiting the formation of the mitotic spindle apparatus and the mitosis process [184,185,186].

Due to the scarcity of *T. brevifolia* bark, the massive exploitation of the Pacific yew was prohibited by an international convention. Consequently, research efforts shifted toward finding other plant sources capable of synthesizing paclitaxel or its biosynthetic precursors. The European yew (*Taxus baccata*), for instance, produces a paclitaxel biosynthetic intermediate (baccatin III) that can be extracted from its removable needles and chemically converted into taxol [187]. Since then, paclitaxel has been reported to be present in several non-*Taxus* plants, such as hazel trees (*Corylus avellana*). It can also be produced in liquid cultures of meristematic *Taxus* plant cells [188]. The extraction, purification, and chemical modification of paclitaxel are among the most notable achievements in pharmaceutical biotechnology, although these processes are not discussed in this article.

The interest switched then to the investigation of filamentous fungi that may produce paclitaxel. A breakthrough occurred when the endophytic fungus *T. andreanae* was isolated from the bark of *T. brevifolia* [61,189]. Subsequent studies investigated paclitaxel-producing endophytic fungi in various *Taxus* species and even in unrelated plant genera [190,191]. Numerous ascomycete fungi isolated from plants were found to produce paclitaxel, and within a few years, several fungi were reported as paclitaxel producers, reaching nearly 200 species, including *Pestalotiopsis versicolor* [192] and *Aspergillus* spp. [193]. Interestingly, marine bacteria associated with algae have also been reported to produce this molecule [194]. However, in many cases, the yield of paclitaxel from fungal sources is extremely low, typically only a few micrograms per liter. This has led to criticism regarding the reliability of the reported compound, mainly due to insufficient confirmation by NMR spectroscopy [62]. In addition, many strains lost paclitaxel production upon repeated subculturing, and most studies did not use isotope-labeled precursors, suggesting that the compound detected in earlier studies may have originated from the accumulation of plant-derived paclitaxel rather than true de novo synthesis by the fungi [195].

Further skepticism arose because some reported paclitaxel-producing fungi lack genes homologous to those involved in the paclitaxel biosynthetic pathway in *Taxus* plants. Although it remains possible that these fungi employ different enzymatic mechanisms to synthesize paclitaxel or paclitaxel-like molecules, this issue continues to be debated [62]. Over the past decades, significant research has focused on identifying fungal genes that encode enzymes homologous to those involved in paclitaxel biosynthesis in *Taxus* plants. It is estimated that the complete biosynthetic pathway in plants requires 19 enzymatic activities, although some of these enzymes remain uncharacterized. A putative paclitaxel biosynthetic pathway has been proposed based on several complementary approaches, including (i) studies in *Taxus* suspension cell cultures, (ii) analysis of *Taxus* cDNA libraries in paclitaxel-producing cells and their expression in yeast systems, (iii) investigations of paclitaxel-producing *Taxus* endophytic fungi, (iv) studies on known intermediates used in semisynthetic paclitaxel production, (v) comparisons with biosynthetic pathways of related terpenoid products, and (vi) insights from synthetic biology efforts, among others [190,196,197,198,199]. Although the emerging pathway provides a detailed framework, it remains incomplete, and only a subset of the enzymes involved has been fully characterized.

Paclitaxel is composed of two distinct moieties formed through separate biosynthetic routes. The first one is a C20 terpenoid moiety, taxadiene, synthesized via the cyclization of geranylgeranyl diphosphate catalyzed by the taxadiene synthase. Taxadiene is then hydroxylated at C10 and C13 by β-C10 and α-C13 hydroxylases, respectively, and the resulting hydroxyl groups are subsequently acylated by specific acyltransferases. The second fragment originates from the phenylpropanoid pathway. Here, phenylalanine is isomerized to β-phenylalanine by the phenylalanine amino mutase, then hydroxylated to produce β-phenylisoserine. This compound is activated via CoA thioester formation by a phenylisoserine-CoA transferase, as commonly occurs in early steps of the phenylpropanoid pathway [200]. In its activated form, this phenylisoserine-CoA is attached to the taxadiene core by a phenylpropanoid acyltransferase, a key enzyme in paclitaxel biosynthesis [190].

Based on plant-derived paclitaxel biosynthetic information, multiple attempts have been made to identify homologous genes in reported paclitaxel-producing fungi. These efforts have included PCR using primers designed from plant biosynthetic genes, as well as RT-PCR amplification from fungal mRNA to obtain cDNA [190] and expression in yeasts. However, the identification of the paclitaxel pathway in most filamentous fungi remains elusive. Putative paclitaxel biosynthetic enzymes have been identified only in endophytic fungi isolated from *Taxus* species, and are encoded by genes homologous to those found in plants [201,202]. Nonetheless, there is no conclusive evidence of a complete paclitaxel biosynthetic pathway in any fungal species to date. Enzymes found in non-endophytic, paclitaxel-producing filamentous fungi, may participate in paclitaxel biosynthesis using non-conventional pathways, or they may be involved in the production of other secondary metabolites and have been mistakenly considered as enzymes of paclitaxel biosynthesis. For instance, the oxygenases, hydroxylases, and acylases present in paclitaxel-producing fungi may exhibit similarities to homologous enzymes involved in the biosynthesis of different terpenoids or flavonoids [190].

Regarding evolution of endophytic fungi isolated from *T. brevifolia* or *T. baccata*, it has been suggested that some genes may have been transferred from the host plant to the fungi. However, the key question of why the transferred genetic material consists of isolated genes rather than the complete biosynthetic pathway still remains unresolved. Moreover, paclitaxel production is not limited to *Taxus* species, as it has also been reported in non-*Taxus* plants such as hazel. Interestingly, the phenylalanine amino mutase putatively involved in paclitaxel biosynthesis in the endophytic fungus *Penicillium aurantiogriseum*, isolated from hazel, shares only 43% sequence identity with the corresponding enzyme of the hazel plant. This raises the question of whether this enzyme is truly part of the paclitaxel biosynthetic pathway, or if it functions in a distinct secondary metabolite pathway [203]. In summary, it remains uncertain whether paclitaxel biosynthesis in fungi proceeds through the same pathway as in *Taxus* plants, or whether only certain steps, such as taxadiene formation, are shared. Further molecular and genetic studies are required to elucidate the precise steps involved in fungal paclitaxel biosynthesis.

Other diterpenoid compounds with potential antitumor activity are produced by fungi. For example, the α-pyrones **higginsianins A** and **B** (Figure 7) were isolated from the mycelial cultures of the fungal pathogen *Colletotrichum higginsianum* grown in liquid medium, and demonstrated potent antiproliferative activity across six cancer cell lines, including Hs683 (oligodendroglioma), U373 (glioblastoma), A549 (non-small cell lung carcinoma), MCF-7 (breast carcinoma), SK-MEL-28 (melanoma), and B16F10 (murine melanoma) [204,205].

#### 3.3.3. Triterpenes

For decades, basidiomycetes have been recognized as an excellent source of natural products and have been widely studied in the search for antitumor agents [206].

**Figure 8:**
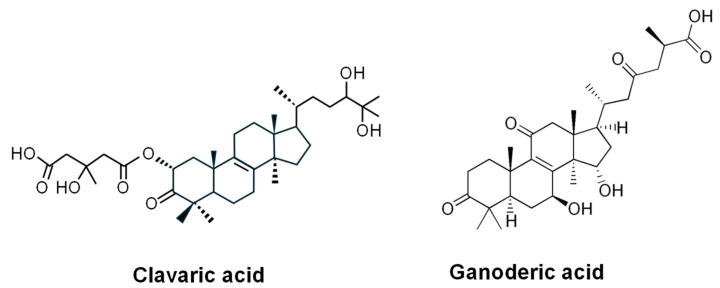
Chemical structure of the antitumor fungal triterpenes clavaric acid and ganoderic acid.

**Clavaric acid** (Figure 8) was discovered in a mushroom later identified as *Hypholoma* (*Naematoloma*) *sublateritium*, during a search for inhibitors of the human RAS prenyltransferase [207,208]. This compound functions as an inhibitor of human RAS-prenyltransferase, an enzyme that modifies the RAS protein and is involved in the development of several solid tumors [209]. Prenylation of RAS by Ras-prenyltransferases plays a key role in tumor initiation and progression. Subsequently, the antitumor activity of *H. sublateritium* was studied. Using hexane extracts of this fungus, inhibition of TNF-α-induced metastasis in MDA-MB-231 breast cancer cells was observed [210]. Furthermore, butanol extracts of this fungus have been shown to possess potent anti-inflammatory activity [211]. A more recent report extended these findings, showing that extracts inhibited pro-inflammatory proteins such as cyclooxygenase-2 (COX-2), cytosolic prostaglandin E2 synthase (cPGES), and the antioxidant nuclear factor erythroid 2-related factor 2 (Nrf2) [212].

**Figure 9:**
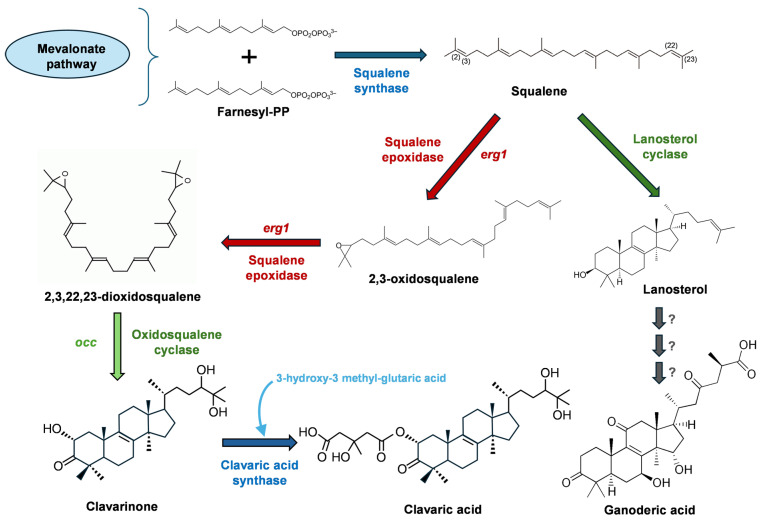
Clavaric acid and ganoderic acid biosynthetic pathways. Numbers in the squalene molecule indicate the site for subsequent oxidations. Question marks in the ganoderic acid biosynthetic pathway denote hypothetical oxidation, reduction, and acetylation reactions.

The biosynthetic pathway of clavaric acid is related to that of the primary metabolite lanosterol, which is a precursor of **ganoderic acid** (Figure 8). Starting from the triterpenoid squalene, the biosynthesis of clavaric acid (Figure 9) requires oxidation to 2,3-oxidosqualene and then to 2,3,22,23-dioxidosqualene. These two initial reactions are catalyzed by the flavoprotein squalene epoxidase, encoded by the *erg1* gene [213]. This gene, cloned from *H. sublateritium*, encodes a protein requiring FAD, NADPH, and P450 cofactors for activity. Overexpression of *erg1* led to transformants of *H. sublateritium* with increased clavaric acid production (32–97% higher than the parental strain, depending on the transformant). In contrast, silencing of *erg1* via antisense RNA reduced clavaric acid production and generated a strain requiring ergosterol for full growth. These findings indicate that *erg1* participates in both clavaric acid and ergosterol biosynthesis through common steps in a branched pathway [207,208]. The *erg1* gene has also been cloned from other fungi, including *Trichoderma harzianum*, which produces trichothecenes and other secondary metabolites, but not clavaric acid. In *T. harzianum*, silencing of *erg1* affected ergosterol biosynthesis [214].

After the formation of 2,3,22,23-dioxidosqualene, the biosynthetic routes to ergosterol and clavaric acid diverge. In ergosterol biosynthesis, oxidosqualene is cyclized to lanosterol by oxidosqualene–lanosterol cyclase (OSLC), which catalyzes internal cyclization to yield a molecule with seven stereospecific carbon atoms [215]. In *H. sublateritium*, however, this cyclization instead yields clavarinone, a late intermediate in clavaric acid biosynthesis. The *occ* gene, encoding oxidosqualene–clavarinone cyclase (OSCC), was cloned by Godio and Martín [216]. Deletion of this gene abolished clavaric production, confirming its essential role in clavaric acid biosynthesis. Interestingly, *occ*-defective mutants did not require sterols, suggesting that this enzyme catalyzes a separated branch for clavaric acid biosynthesis. Amplification of the *occ* gene increased clavaric acid production by 35–67% compared to the parental strain, depending on the transformant. Analysis of the OSCC protein showed similarity to other oxidosqualene cyclases, such as OSLC, but with key differences in amino acids at the enzyme’s active site [216]. OSCC was modeled in comparison with the crystal structure of human oxidosqualene cyclase [217] and visualized with the DeepView–Swiss Pdb Viewer software (version 4.1) [218]. The model revealed a large cavity corresponding to the oxidosqualene cyclization cleft, surrounded by β-sheets and α-helices forming a hollow barrel structure. Within this cavity lies the conserved VSDCXXE motif (amino acids 448–454), which forms part of the active site of oxidosqualene cyclases. The presence of a specific OSCC in *H. sublateritium*, distinct from OSLC, is consistent with findings in other basidiomycetes, such as *Coprinus cinereus* and *Laccaria herbicola*, which also contain two oxidosqualene cyclases. In these fungi, as well as in *H. sublateritium* and in plants, one of the cyclase genes is linked to an actin-related gene (*c-actin*). Similarly, in *Arabidopsis thaliana*, a separate gene cluster for talianol biosynthesis has been identified, which is different from the oxidosqualene–lanosterol cyclase cluster [219]. Altogether, the evidence suggests that fungi and plants share analogous branched pathways for the biosynthesis of squalene-derived compounds.

Conversion of clavarinone to clavaric acid proceeds through the incorporation of a 3-HMG moiety into the sterol nucleus. HMG, an intermediate of the mevalonate pathway, is synthesized by condensation of acetoacetyl-CoA with acetyl-CoA, which is catalyzed by HMG-CoA synthase in both prokaryotes and eukaryotes [220,221,222]. The mechanism of attachment involves linking the carboxyl group of HMG-CoA to a hydroxyl group in clavarinone, with release of CoA. Further studies are required to fully characterize this mechanism.

In recent years, antiSMASH analyses have identified clusters of genes in the genomes of various filamentous fungi, basidiomycetes, and ascomycetes that have been proposed to encode clavaric acid biosynthetic enzymes because they contain a squalene cyclase. These include *Cordyceps pseudotenuipes* [223], *Aspergillus brasiliensis* [224], four species of *Sporothrix* [225], four species of *Penicillium* [226], *Pycnoporus sanguineus* [227], and a marine isolate of *A. terreus* [228]. The genes in these clusters are similar to one another, but the squalene cyclases they encode share only 48–50% sequence identity with the experimentally confirmed enzyme of *H. sublateritium*. Moreover, the surrounding genes in these clusters differ completely from those near *H. sublateritium occ*. Importantly, none of these genome studies have provided experimental evidence of clavaric acid production in the sequenced strains, and thus, the presence of clavaric acid biosynthetic genes in these fungi should be interpreted with caution.

Another antitumor triterpenoid related to ergosterol that derives also from squalene is **ganoderic acid** (Figure 8), which is produced by the basidiomycete *Ganoderma lucidum*. Ganoderic acid inhibits lung cancer metastasis and induces tumor apoptosis, exhibiting cytotoxic effects on various cancer cell lines. It also shows antiviral (anti-HIV) activity [229,230]. Ganoderic acid is synthesized from squalene via lanosterol (Figure 9). The initial step of the pathway is similar to that involved in clavaric acid [231]. In the last steps of the biosynthetic pathway lanosterol is transformed to ganoderic acid by oxidation, reduction and acetylation [232]. The biosynthetic genes have been identified in the *G. lucidum* genome [233]. Other triterpenes from this fungus, such as lucidimols, ganodermanondiol, and ganoderiol F, have shown anticancer activities [234].

Different triterpenoids and sesquiterpenoids from the mushroom *Antrodia camphorata*, including antroquinonol, 4-acetylantroquinonol B, and antroquinonol D, exhibited anticancer effects against colon, lung, liver, breast, and glioblastoma cancer cells [235].

#### 3.3.4. Meroterpenoids

The meroterpenoid family comprises compounds that contain a fifteen-carbon isoprenoid unit (i.e., farnesyl unit) linked to a tetraketide-derived orsellinic acid molecule [236].

**Figure 10:**
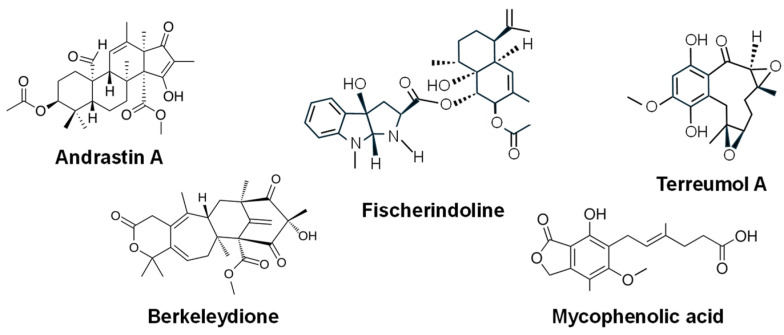
Chemical structure of some fungal meroterpenoids with antitumor activity.

Among the members of this family, andrastins A–D were identified in a *Penicillium chrysogenum* strain during a screening program for antitumor compounds at the Kitasato Institute (Japan) in the group of Nobel price S. Omura [237,238,239]. They are also produced by *P. roqueforti* during the maturation of blue cheeses, where they accumulate in large amounts, particularly in Cabrales, a roquefort type cheese [240,241,242]. **Andrastin A** (Figure 10) displays strong antitumor activity by inhibiting human RAS prenyltransferase, an enzyme essential for the transformation of normal human cells into tumor cells [238,239]. Andrastin A has also been shown to inhibit the efflux of anticancer drugs in multidrug-resistant tumor cells, enhancing their intracellular accumulation [243].

Other meroterpenoids include **terreumol A** (Figure 10), a rare 10-membered-ring molecule isolated from the fruiting bodies of the mushroom *Tricholoma terreum*. It provided notable anticancer activity against different cancer cell lines, such as MCF-7 breast cancer, A-549 lung cancer, and SMMC-7721 hepatocellular carcinoma, human myeloid leukemia HL-60 and colon cancer SW480 [244]. **Mycophenolic acid** (Figure 10), which was initially defined as an antimicrobial drug, but it was soon described as both an antitumoral and immunosuppressive agent [16], is another meroterpenoid composed of a PK-derived phthalide core linked to a farnesyl-like terpenoid side chain. It is produced by different *Penicillium* species [245] and showed cytotoxicity against several cancer cell lines and significantly inhibited tumor growth in vivo [246]. **Berkeleydione** (Figure 10), isolated from an extremophilic acid-tolerant *Penicillium* sp. residing in the highly acidic waters of Berkeley Pit Lake in Butte, Montana, exhibited antitumor activity in vitro, particularly against non-small-cell lung cancer cell lines [247]. **Fischerindoline** (Figure 10) is a terpenoid pyrroloindole obtained from both solid and liquid cultures of the ascomycete fungus *N. pseudofischeri* that demonstrated growth inhibitory effects across a panel of several cancer cell lines, including A549 (non-small cell lung cancer), SK-MEL-28 (melanoma), Hs683 (oligodendroglioma), U373 (glioblastoma), MCF7 (breast cancer), and B16F10 (murine melanoma) [248].

**Figure 11:**
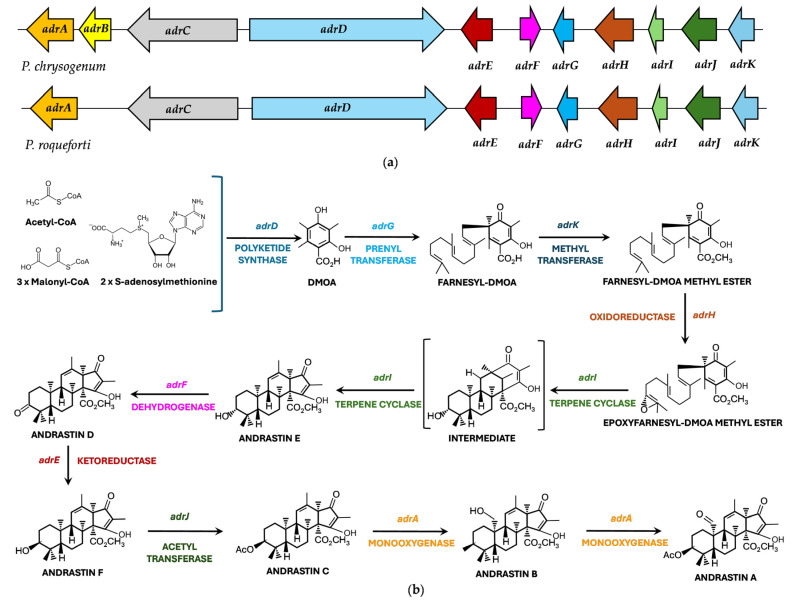
Biosynthesis of andrastin A: (**a**) Andrastin A biosynthetic gene cluster in *P. chrysogenum* and *P. roqueforti*. Note the partial absence of the *adrB* gene in *P. roqueforti*; (**b**) Proposed biosynthetic pathway for andrastin A in *P. roqueforti*. DMOA (3,5-dimethylorsellinic acid). Adapted from [249,251,255].

Studies on the biosynthesis of austinol and other meroterpenoids provided the bases to elucidate the biosynthesis pathway of andrastins. It was shown in *P. chrysogenum* that andrastins derive from a tetraketide formed by the PKS AdrD, which is cyclized to form 3,5-dimethylorsellinic acid (DMOA) [249]. This compound is subsequently linked to a C15 epoxyfarnesyl isoprenoid moiety by a prenyltransferase. These authors identified an eleven-gene cluster, the *adr* cluster, and using expression tools, subcloned five genes (*adrI*, *adrF*, *adrE*, *adrJ* and *adrA*) in *Aspergillus oryzae*, successfully characterizing several pathway intermediates, namely andrastins E, D, F, C, B, and A. The *adr* cluster in *P. chrysogenum* (Figure 11), includes genes for AdrD, a PKS (tetraketide synthetase); AdrG for a prenyltransferase (that attaches a farnesyl group to DMOA); AdrH, for a FAD-Monooxygenase; AdrJ for a methyltransferase; and AdrI, for a terpene cyclase that cyclizes epoxyfarnesyl-DMOA methyl ester to andrastin E. In addition, the *adrE*, *adrF*, *adrJ*, and *adrA* genes encode “decorating” enzymes (ketoreductase, short-chain dehydrogenase, acetyltransferase, P450 monooxygenase), whereas *adrC* encodes an MFS transporter. Only *adrB*, encodes a protein with unclear or accessory functions [249]. Following the publication of the *P. roqueforti* FM164 genome sequence [250], the andrastin gene cluster in *P. roqueforti* CECT2905 (Figure 11), a strain originally isolated from a Roquefort-type cheese, was described [251]. The gene cluster of *P. roqueforti* CECT2905 shares 99% identity with the andrastin cluster in the FM164 genome sequence, indicating that the andrastin gene is conserved among *P. roqueforti* strains isolated from blue-veined cheeses in different countries [252]. The encoded proteins in *P. roqueforti* CECT2905 exhibit 70–90% identity with those from *P. chrysogenum*. Compared to *P. chrysogenum*, the *P. roqueforti* cluster shows evidence of rearrangement and partial gene deletion of the *adrB* gene, reflecting the evolutionary divergence of these two *Penicillium* species from a common ancestor. A similar gene-loss rearrangement event has been reported in the roquefortine/meleagrin gene cluster in *P. roqueforti*, which give raise to a natural mutant unable to convert roquefortine to meleagrin [144]. Involvement of all nine *adr* genes in andrastin A biosynthesis was confirmed by functional validation through antisense RNA-mediated silencing, and a biosynthetic pathway was proposed [251] (Figure 11). Interestingly, silencing of the *adrC* gene (encoding an MFS transporter) had no drastic effect on secretion of andrastins. While MFS-type transporters are often assumed to mediate secretion of secondary metabolites, recent studies in filamentous fungi suggest broader roles, including localization in peroxisomal membranes or vesicular compartments. These transporters may contribute to intracellular traffic, sequestration, or compartmentalization of intermediates via vesicle-mediated systems (“toxisomes”) [125,253,254].

### 3.4. Phenolic Compounds

**Figure 12:**
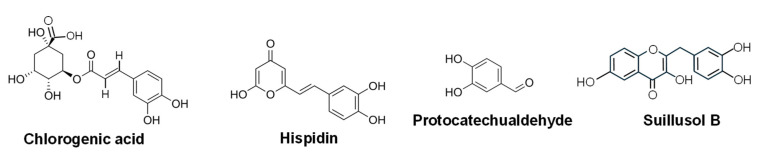
Chemical structure of some phenolic compounds with antitumor activity from non-endophytic fungi.

**Hispidin** (Figure 12) is a phenolic PK styrylpyrone found in various fungi (e.g., *Inonotus* and *Phellinus* species) that shows cytotoxicity in different cancer cell lines [256] and induces apoptosis in human colon cancer cells via ROS-mediated intrinsic and extrinsic pathways [257].

**Chlorogenic acid** (Figure 12) is a polyphenol ester composed of caffeic acid and quinic acid that belongs to the family of hydroxyl cinnamic acid esters biosynthesized by *Sclerotium rolfsii*, a soil-borne phytopathogenic fungus. It induces apoptosis in numerous cancer cell lines including colon, breast and lung, inhibiting metastasis and improving antitumor immunity in breast cancer [258].

**Protocatechualdehyde** (Figure 12), also known as 3,4-dihydroxybenzaldehyde or Rancinamycin IV, is a simple phenolic aldehyde produced by the medicinal mushroom *Phellinus gilvus* that induces G0/G1 phase arrest and apoptosis in murine B16-F10 cells [259], inhibits proliferation and induces apoptosis in human breast cancer cells [260], in addition to showing antitumor activity in human colorectal cancer cell lines [261].

**Suillusol B** (Figure 12), a polyphenolic metabolite isolated from *Suillus granulatus*, exhibited cytotoxic effects against the HepG2 liver cancer cell line [262].

### 3.5. Other Compounds

**Figure 13:**
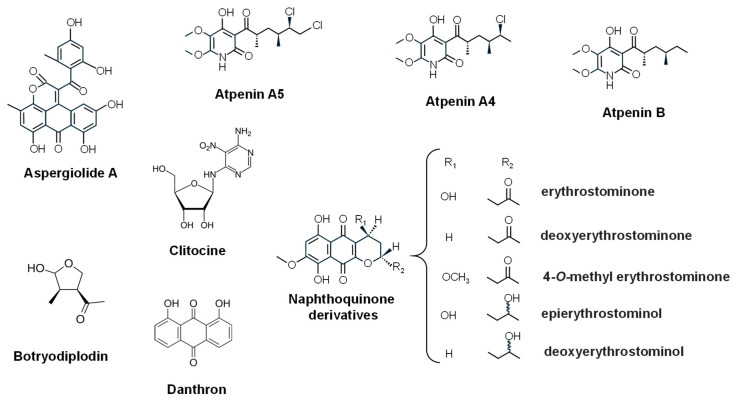
Chemical structures of several structurally diverse fungal antitumour compounds.

Atpenins are 2-pyridones featuring a substituted pyridine ring system with unique functional-group patterns, notably including chlorinated forms (e.g., A4, A5). They were originally isolated in 1988 from a soil-derived strain of *Penicillium* sp. FO-125 [263]. More recently, two new atpenins, NBRI23477 A and B, were obtained from the fermentation broth of *Penicillium atramentosum* PF1420 isolated from a soil sample in Japan, and together with **atpenins A4**, **A5**, and **B** (Figure 13) inhibited the growth of human prostate cancer DU-145 cells more effectively in coculture with human prostate stromal cells than in monoculture [264].

**Botryodiplodin** (Figure 13) features a lactone (cyclic ester) ring and a substituted tetrahydrofuran moiety, initially isolated from the plant pathogen *Lasiodiplodia theobromae* (*Botryodiplodia theobromae*) [265] and produced by different fungi, including *P. roqueforti* [266] and the pathogenic fungus *Macrophomina phaseolina* [267]. This compound exhibited activity against different cancer cell lines, including leukemia, sarcoma, and cervical cancer [65,268].

Naphthoquinones are interesting molecules that has antitumor activity. Several naphthoquinone derivatives are produced by the ant pathogenic fungus *Ophiocordyceps unilateralis*, including **erythrostominone**, **deoxyerythrostominone**, **4-O-methyl erythrostominone**, **epierythrostominol** and **deoxyerythrostominol** (Figure 13), which exhibited cytotoxicity against BC, KB and Vero cell lines [269]. Naphthoquinone-related PKs are also produced by fungi living inside healthy lichen tissues (endolichenic fungi). For example, the spirobisnaphthalene plecmillin A isolated from the fungus strain (CGMCC 3.15192), and endolichenic fungus derived from *Peltigera elisabethae* var. mauritzii, exhibited potent growth inhibitory effect on colon HCT116 and osteosarcoma U2OS cancer cell lines by inducing both cell cycle arrest at the G2/M phase and cell death [270].

**Clitocine** (Figure 13), a fungal nucleoside isolated from the mushroom *Clitocybe inversa* [271] and *Leucopaxillus giganteus* demonstrated anti-proliferative effects against human cervical cancer HeLa cells primarily by inducing apoptosis [272].

Several anthraquinone derivatives have been identified from marine-derived fungi [273], some of them showing antitumor activity. For example, **1,8-dihydroxy-9,10-anthraquinone (danthron)**, isolated from the marine fungus *Chromolaenicola* sp. has antiangiogenic and antitumor against human breast carcinoma MDA-MB231 and fibrosarcoma HT1080 cell lines [274]. **Aspergiolide A** (Figure 13) is an anthraquinone-derived PK isolated from the marine-derived fungus *Aspergillus glaucus* HB1-19 [275] that has demonstrated selective cytotoxicity against multiple human lung, leukemia, and hepatocellular carcinoma cancer cell lines [276].

## 4. Conclusions

Over the past few decades, particularly since the latter part of the twentieth century, tremendous efforts have been made in laboratories around the world to discover new antitumor agents. This is largely because cancer, in its various forms, remains one of the leading causes of human mortality. Researchers have explored a wide range of living organisms—including plants, filamentous fungi, and bacteria—in the search for compounds with anticancer activity, leading to significant advances in the isolation of diverse classes of anticancer molecules. Many Basidiomycetes are well known for their traditional use as food and for their bioactive natural products utilized in folk medicine [277]. Moreover, the number of mushroom species with available genome sequences has increased significantly. In addition, the study of endophytic fungi from plants other than *Taxus* spp. has gained considerable interest, as these organisms represent a promising source of novel secondary metabolites with potential antitumor properties [58,59,60].

In this article, we reviewed the contribution of different fungi to the production of diverse secondary metabolites with antitumor activity. The most representative compounds are included in Table 1. Several promising fungal-derived antitumor agents have already progressed to clinical evaluation. For example, the phenylahistin derivative plinabulin has entered clinical trials [113,114,115]. Likewise, certain statins—such as lovastatin and mevastatin—originally developed as cholesterol-lowering agents have demonstrated apoptosis-related inhibition of tumor cell proliferation and enhancement of the efficacy of other chemotherapeutic agents in both preclinical and clinical studies [70,72].

Despite the multiple in vivo and in vitro studies demonstrating the antitumor effects of fungal-derived compounds, their advancement toward therapeutic applications depends critically on understanding their pharmacokinetic properties, toxicity, and production feasibility (Table 2). Many of these compounds show promising cellular activities, although they lack in vivo studies to assess their safety and metabolic behavior. Furthermore, their complex biosynthetic pathways and low yields hinder large-scale production, driving interest in synthetic biology and metabolic engineering strategies to optimize their production and generate more suitable analogues for pharmaceutical development.

The diversity of filamentous fungi that produce such compounds—including both Basidiomycetes and Ascomycetes—is remarkable, with over one hundred fungal species reported as producers of antitumor molecules. Notably, yeasts, which possess relatively small genomes, are not known to produce antitumor agents. This observation reflects a well-established fact: the absence of secondary metabolite biosynthetic gene clusters in the best-studied yeasts. The lack of these gene clusters is not due to the absence of mycelial development, but rather to evolutionary gene loss. During evolution, yeasts such as *S. cerevisiae* and *Candida albicans* have adapted to stable habitats with limited nutrient variation, which has reduced the selective pressure to maintain genes involved in secondary metabolite biosynthesis. Consequently, these yeasts do not require secondary metabolites for defense against microbial competitors or for resistance to biotic and abiotic stress. However, it remains possible that some rare, yeast-like fungi—still poorly characterized at the biochemical level—may retain secondary metabolite gene clusters and thus, represent potential sources of novel bioactive compounds. The impressive diversity of antitumor molecules produced by filamentous fungi provides a solid foundation for further characterization and development of novel anticancer agents. Although many of the isolated compounds exhibit significant cytotoxicity against cancer cells, they often also display some degree of toxicity toward normal human cells. Therefore, extensive research is still required to assess and improve the selective toxicity of candidate molecules in order to minimize adverse effects and reduce the likelihood of rejection during clinical trials.

One of the most remarkable successes in the discovery of antitumor agents from natural sources is paclitaxel, which remains widely used in the treatment of several solid tumors despite the limited availability of *Taxus* bark. This scarcity has prompted extensive efforts to identify filamentous fungi capable of producing paclitaxel, and numerous endophytic and non-endophytic fungal species have been reported as putative producers. Although the original 1993 report of paclitaxel production by the endophytic fungus *T. andreanae* sparked widespread interest and over 200 subsequent claims of fungal producers, the issue remains controversial. Early studies relied on indirect analytical methods such as immunoassays, HPLC, and mass spectrometry to detect paclitaxel in fungal cultures, often yielding very low and unstable amounts and lacking rigorous structural confirmation, which raised concerns about reproducibility and the possibility of analytical artifacts or plant-derived contamination. More recently, “hyper-production” of paclitaxel (up to 1.6 g/L) in an endophytic *A. fumigatus* strain isolated from *Taxus* sp. was reported [193], and the presence of the *dbat* gene, encoding 10-deacetylbaccatin III 10-O-acetyl transferase (a key enzyme in the paclitaxel biosynthetic pathway), was confirmed. Nevertheless, the presence of *dbat* alone does not indicate the existence of a complete biosynthetic pathway, as paclitaxel production in plants requires multiple additional enzymes (e.g., taxadiene synthase, several P450s, and acyl transferases) that have not been identified in fungal genomes. Thus, while certain endophytic fungi can produce or accumulate taxane-like metabolites under specific laboratory conditions, genomic and transcriptomic analyses of most claimed fungal producers consistently fail to reveal the full complement of biosynthetic genes. Therefore, a complete characterization of the biosynthetic gene cluster or pathway responsible for its production is necessary to confirm the ability of fungi to produce this anticancer compound. In the meantime, the biosynthesis of paclitaxel—or its intermediates—in fungi remains highly debated, with ongoing discussions about the reliability of certain published data [62]. In our opinion, true, autonomous production of paclitaxel remains a unique feature of *Taxus* plants, and reports of fungal-derived paclitaxel likely reflect partial pathways, elicitor-induced accumulation of intermediates, or analytical overestimation rather than bona fide, de novo paclitaxel biosynthesis. To clarify these uncertainties, further molecular, genetic, and biochemical studies are needed, and the advent of modern molecular tools, including next-generation sequencing, greatly facilitates the identification of putative paclitaxel biosynthetic gene clusters and other pathways involved in the production of antitumor compounds.

Overall, fungi continue to offer tremendous potential for the discovery of novel anticancer molecules.

## Figures and Tables

**Table 1:** Most representative fungal compounds with antitumor activity and preclinical and clinical status.

Compound	Chemical Class	Producing Fungi	Antitumor Activity/ Preclinical and Clinical Status
Andrastin A	Meroterpenoid	*P. chrysogenum*	RAS-prenyltransferase inhibitor. It blocks the efflux of anticancer drugs/ Preclinical stage (in vitro studies) [238,239,241]
		*P. roqueforti*	
Asperpyrone A/B	Dimeric naphthopyrones (aromatic PK)	*Aspergillus* sp. XNM-4	Cytotoxic to SK-OV-3 and PANC-1/ Preclinical stage (in vitro studies) [74]
Brefeldin A	Macrolide PK	*Paecilomyces*, *Alternaria*, *Curvularia*, *Penicillium*, *Phyllosticta*	Cytotoxic to HL-60, KB, HeLa, MCF-7, Spc-A1. It disrupts Golgi function/ Preclinical stage (in vivo study with subcutaneous and subrenal capsule melanoma mouse models) [75,76,77,78,278]
Clavaric Acid	Triterpene	*H. sublateritium*	RAS-prenyltransferase inhibitor; anti-metastatic activity/ Preclinical stage (in vitro studies) [207,208,209]
Fumitremorgin C	DKP alkaloid	*A. fumigatus*	Selective BCRP/ABCG2 inhibitor (overcomes multidrug resistance)/ Preclinical stage (in vitro studies) [119]
Gliotoxin	DKP alkaloid	*A. fumigatus*, *N. pseudofischeri*, *T. virens*, *D. cejpiii*	Highly active across breast, colorectal, leukemia, lung cell lines. Potent apoptosis induction via mitochondrial pathway activation of Bax, caspases, and cytochrome c release/ Preclinical stage (in vivo study with rat mammary carcinoma model [134,135,136,279]
Griseofulvin	Chlorinated PK	*P. griseofulvum* and other ascomycetes	Broad antitumor activity: breast, lung, colorectal, cervical cancers. Microtubule inhibition, mitotic arrest/ Approved as antifungal drug, but not established as chemotherapy. Preclinical stage (in vivo studies with nude mouse xenograft models of small-cell lung cancer) [84,85,86]
Lovastatin	PK	*A. terreus*, *P. citrinum*, *M. ruber*	Anticancer activity across many tumors. It enhances chemotherapy responses. Apoptosis induction, cell-cycle arrest, angiogenesis inhibition/ Approved as a lipid-lowering drug. Preclinical in vitro and in vivo evidence for anticancer effects and preclinical and clinical/epidemiological evidence of synergy with other drugs, but not approved for cancer treatment [70,72]
Paclitaxel (Taxol^®^)	Diterpene	Endophytic fungi (e.g., *T. andreanae*)	Microtubule-stabilizing agent that blocks mitosis/ Clinically approved anticancer drug [280]
Phenylahistin	DKP alkaloid	*A. ustus*	Tubulin inhibitor; strong cytotoxic activity/ Parent of clinical candidate plinabulin (Phase III study in prophylaxis of chemotherapy-induced neutropenia) [110,111,112,113,114,115]

**Table 2:** Overview of ADME, toxicity, production constraints and improvement prospects of the most representative fungal compounds with antitumor activity.

Compound	ADME Studies	In Vivo Toxicity	Production/ Improvement Prospects
Andrastin A	Not available	Not reported	Low yield/ Heterologous production of andrastin A by the expression of the biosynthetic gene cluster in *A. oryzae* [249]
Asperpyrone A/B	Not available	Not reported	Low yield/ Not reported
Brefeldin A	Poor oral availability. Rapid elimination in mice [281,282]	Significant toxicity [283]	Moderate yield, but complex purification/ Synthesis of more stable and less toxic analogs with improved bioavailability [284,285]
Clavaric Acid	Not available	Not reported	Low yield/ Overepression of biosynthetic genes in *H. sublateritium* [213,216]
Fumitremorgin C	Widely distributed to tissues (two-compartment model), including brain and tumor. Hepatic metabolism as a primary mechanism of elimination [286]	Tremorgenic activity (mycotoxin) [287]. No severe toxicity reported after i.v. administration to mice [286]. Neurotoxicity reported for some animals [288]	Low yield/ Development of synthetic analogs (Ko143 and its analogs K2 and K34) with relatively improved toxicity profile and favorable oral pharmacokinetic profiles [289,290]
Gliotoxin	Not available	Pronounced immunosuppressive effects and potential hepatotoxicity [291,292]	Low yield, complex purification/ Overexpression of transcription factors in *A. fumigatus* [293,294,295]
Griseofulvin	Well-studied (approved antifungal drug). Poorly absorbed from the gastrointestinal tract [83]	Headaches, gastrointestinal reactions and cutaneous eruptions in humans [296]. Liver and thyroid cancer in rodents. Teratogenicity, and embryotoxicity in various species [297]	High titers produced under controlled submerged fermentation using industrial strains [298]
Lovastatin	Well-characterized (approved lipid-lowering drug). Low oral absorption and limited bioavailability, with dependence on hepatic metabolism for activation [299]	High doses in animals can produce significant toxicity, including hepatic and renal necrosis in rabbits [300,301]. Possible off-target effect by altering dietary lipid absorption in rats [302]	Highly optimized industrial process, with high yields achieved using *A. terreus/* Metabolic engineering strategies have been widely applied to significantly increase lovastatin titers in fermentation [303,304]
Paclitaxel (Taxol^®^)	Well-characterized. Extensive tissue distribution, high plasma protein binding, minimal renal elimination, extensive hepatic metabolism [305]	Hematopoietic suppression, lymphoid organ atrophy, and reproductive organ effects at high doses in rats [306,307]. Systemic toxicity associated with its solubilizing agent cremophor EL [308]	Optimized process using semi-synthetic precursors (e.g., baccatin III) or plant-cell suspension cultures of *Taxus* sp. [188]/ Fermentation with the paclitaxel-producing endophyte *A. fumigatus* has been proposed as promising alternative [193]
Phenylahistin	Not available	Not reported	Low yield/ Development of more potent derivatives such as plinabulin (NPI-2358) [309]

## Data Availability

No new data were created or analyzed in this study. Data sharing is not applicable to this article.

## Abbreviations

The following abbreviations are used in this manuscript:

DKP	Diketopiperazine
DMOA	3,5-dimethylorsellinic acid
HMG	Hydroxy-3-methylglutaryl
NRP	Non-ribosomal peptides
NRPS	NRP synthetase
OSCC	Oxidosqualene–clavarinone cyclase
OSLC	Oxidosqualene–lanosterol cyclase
PK	Polyketide
PKS	PK synthase
